# Fosfomycin-induced agranulocytosis: a case report and review of the literature

**DOI:** 10.1186/s12879-023-08652-8

**Published:** 2023-10-13

**Authors:** Elodie Matusik, Julien Demanet, Isabelle Alves, Alina Tone, Nicolas Ettahar, Justine Lemtiri, Camille Potey, Sophie Gautier, Fabien Lambiotte, Louise Gaboriau

**Affiliations:** 1 Centre Hospitalier de Valenciennes, Valenciennes, France; 2 Centre Hospitalier Régional et Universitaire de Lille, Lille, France

**Keywords:** Fosfomycin, Agranulocytosis, leucopenia, Neutropenia, Adverse drug reaction, Drug safety

## Abstract

**Background:**

The intravenous form of fosfomycin, a bactericide antibiotic used to treat multiresistant bacterial infections is little prescribed. The most common reported adverse effects are hypokaliemia and hypernatremia. We describe a case of agranulocytosis, a rarely described side effect that may be fatal.

**Case presentation:**

A 45 year-old woman was admitted to the intensive care unit for post-surgical meningitis following meningioma resection. Meropenem and vancomycin were first introduced. A DRESS-syndrom with meropenem was suspected. Neutropenia was diagnosed three days after the introduction of parenteral fosfomycin and agranulocytosis four days later. Eosinophilia was also observed. A bone marrow aspiration was performed showing a disappearance of the neutrophil granulocyte line and a significant eosinophilia. Meropenem was discontinued. Fosfomycin was maintained and filgrastim was added. As filgrastim had no effect, the relationship with fosfomycin was suspected, so it was then withheld. An increase of the neutrophil count was observed. Because of the complexity of the case, the unfavorable course of the illness and the urgent need for revision surgery, a rechallenge with fosfomycin was done followed by a decrease of the neutrophil count.

**Conclusion:**

This is the third paper reporting agranulocytosis induced by fosfomycin, and the first detailed description of a case. Based on chronological and semiological criteria and bibliographic data, the event was qualified as probable with the Naranjo adverse drug probability scale. Literature data is scarce. The summary of product characteristics mentions that only a few cases of transient neutropenia and agranulocytosis have been reported. An analysis of the FDA Adverse Event Reporting System Database highlighted a higher than expected frequency of agranulocytosis in patients treated with fosfomycin. Parenteral fosfomycin is often used in patients receiving other medications, so that it is rarely the only suspect. In our case, the results of the bone marrow aspiration, the sudden drop of the neutrophil count with concomitant eosinophilia and the absence of improvement despite the dose decrease, point towards an immuno-allergic mechanism. However, the overlap between the suspected DRESS induced by meropenem and the agranulocytosis do not allow to conclude with certainty on the causality. Awareness should be raised about this side effect.

**Supplementary Information:**

The online version contains supplementary material available at 10.1186/s12879-023-08652-8.

Fosfomycin is a bactericide antibiotic used against aerobic gram positive and gram negative bacteria. Discovered in 1969, it inhibits bacterial cell wall synthesis by acting on pyruvyl transferase, involved in one of the first steps of peptidoglycan synthesis [1]. The oral formulation of fosfomycin tromethamine is used as a single 3 gram dose in the treatment of uncomplicated cystitis. It is well tolerated with a good safety profile, with gastrointestinal disorders as the most frequent adverse effect [2]. The intravenous form of fosfomycin, available in Europe as the disodium salt, is seldom prescribed [2]. Its use is restricted to the treatment of multiresistant bacterial infections. The recommended dose is 12–16 g/day (up to 24 g/day) divided into 3 to 4 doses. The most common adverse effects reported with parenteral fosfomycin are hypokaliemia and hypernatremia [2, 3]. Agranulocytosis is a rare adverse effect defined by a neutrophil count below 500/mm^3^ which may be fatal. We describe here a case of agranulocytosis associated with parenteral fosfomycin.

## Case summary

A 45 year-old woman was admitted to the intensive care unit (ICU) on December 19th, for post-surgical meningitis following meningioma resection. She had received meropenem and vancomycin since November 26th to treat infection with extended-spectrum beta-lactamases *Enterobacter cloacae* found in the cerebral collection and a catheter infection. Fever persisted despite antibiotics. Cultures from PICC line samples were positive for *Pseudomonas aeruginosa* on December 26th. On December 27th, pruritus and redness of the face and edema of the eyelids were noticed. ASAT and ALAT counts were respectively 187 U/L [N < 67 UI/L] and 167 U/L [N < 67 UI/L]. Normal values were reported on admission in the ICU. The redness and pruritus then spreaded to the whole body. The eosinophil count was 0.30 G/L [N: 0.02–0.58 G/L] on December 25th. An intrinsic renal failure was diagnosed and a vancomycin flushing syndrome was suspected as the patient was treated with high doses of vancomycin in continuous infusion. Vancomycin concentration was 49.1 mg/L on December 27th (eGFR_CKD−EPI_ : 44.5 mL/min/1.73 m²) and 72.7 mg/L on December 29th (eGFR_CKD−EPI_ : 24 mL/min/1.73 m²) with a target concentration > 40 mg/L. Vancomycin was replaced by fosfomycin 4 g QID on December 29th. Neutropenia was diagnosed on January 1st with a neutrophil count falling from 4.18 G/L down to 1.27 G/L [N: 1.40–7.70 G/L] in 17 h, and eosinophils increasing from 1.87 to 2.29 G/L. Total IgE were undetectable. Given the persistence of the erythema, a potential responsibility of the oral ferrous sulphate and pantoprazole was suspected so they were replaced by sodium feredetate and lansoprazole. Serum protein electrophoresis showed an important decrease in albumin levels, compatible with an inflammatory syndrome, and a significant decrease in gammaglobulin levels. A drug reaction with eosinophilia and systemic symptoms (DRESS) was hypothesized considering the skin redness associated with eosinophilia, hepatic cytolysis and renal failure. Fosfomycin was reduced to 4 g/12 h given the renal failure the following day (eGFR_CKD−EPI_ : 26.5 mL/min/1,73 m²). On January 5th, an agranulocytosis was evidenced with a neutrophil count of 0.03 G/L, based on the World Health Organization definition. The eosinophil count was 2 G/L at the time. A bone marrow aspiration was performed showing a disappearance of the neutrophil granulocyte line (0%) and an eosinophilia (44%), without any blasts or cytological abnormality on the megacaryocytic or erythroblastic lines. Meropenem was discontinued and fosfomycin was maintained alone to treat the *Enterobacter cloacae*. Filgrastim was prescribed at the dose of 48 MUI for 5 days. Twenty-four hours later, the eosinophil count was 0.38 G/L and the neutrophil count had gone up to 0.76 G/L. In the following 48 h, the eosinophil count was respectively 0.75 G/L and the neutrophil count dropped down to a nadir of 0 G/L. Lymphopenia was also observed. Fosfomycin was suspected and discontinued on the January 8th. Colistin was added. Given the symptoms of severe sepsis, the delay to get the results of blood cultures, and the dialysis catheter cultures positive for *Candida glabrata and P.aeruginosa*, aztreonam and caspofungin were then started. The neutrophil count improved to 0.22 G/L and 3.69 G/L after 24 and 48 h respectively, as the eosinophil count: 0.82 and 2.08 G/L. After a peak at 2.89 G/L on January 11th, the eosinophil count decreased down to 0.10 G/L on January 23th. Given the persistence of erythema and edema of the lower limbs, aztreonam was discontinued after 7 days of treatment, as was caspofungin 3 days later when the blood cultures came back negative. Because of a delayed healing, a revision surgery was done on January 22th. A rechallenge with meropenem and fosfomycin was discussed three days later because of an abnormal cerebrospinal fluid exam with gram-positive cocci. Meropenem was contra-indicated given the suspicion of DRESS. Fosfomycin on the other hand was restarted even with the suspicion of agranulocytosis given the complexity of the case. The patient received a dose of meropenem by mistake. The day after fosfomycin and meropenem reintroduction (eGFR_CKD−EPI_ : 49 mL/min/1.73 m²), an erythema appeared on the face and the upper limbs associated with eosinophilia. It was imputed to meropenem. Fosfomycin 4 g QID was maintained for 7 days before being replaced by linezolid. A decrease of the neutrophil count was observed to reach a nadir of 1.93 G/L on January 30th, followed by an increase to 3.33 G/L the following day. The agranulocytosis and the DRESS were notified to the regional pharmacovigilance center.

## Discussion

To our knowledge, this is the third paper reporting agranulocytosis induced by fosfomycin, and the first detailed description of a case [4, 5]. The Naranjo adverse drug probability scale was used to evaluate the individual causal relationship between fosfomycin and agranulocytosis. Based on chronological and semiological criteria and bibliographical data, especially the positive rechallenge, the event was qualified as probable (5/10) (Table 1).

**Table 1:** Naranjo score

Question	Yes	No	Do Not Know	Score
1. Are there previous conclusive reports on this reaction?	+ 1	0	0	+ 1
2. Did the adverse event appear after the suspected drug was administered?	+ 2	-1	0	+ 2
3. Did the adverse event improve when the drug was discontinued or a specific antagonist was administered?	+ 1	0	0	+ 1
4. Did the adverse event reappear when the drug was readministered?	+ 2	-1	0	+ 2
5. Are there alternative causes that could on their own have caused the reaction?	-1	+ 2	0	-1
6. Did the reaction reappear when a placebo was given?	-1	+ 1	0	0
7. Was the drug detected in blood or other fluids in concentrations known to be toxic?	+ 1	0	0	0
8. Was the reaction more severe when the dose was increased or less severe when the dose was decreased?	+ 1	0	0	0
9. Did the patient have a similar reaction to the same or similar drugs in any previous exposure?	+ 1	0	0	0
10. Was the adverse event confirmed by any objective evidence?	+ 1	0	0	0
	**Total Score: 5**			

However, the concomitance of a DRESS (possibly induced by meropenem) makes the imputability analysis more complex. The time-to-onset of the reaction, the presence of a IgE-free eosinophilia with a central cause is suggestive of type 4 hypersensitivity. However, no other markers of allergy were tested. Given the risks for the patient, no skin test was realized to strengthen this hypothesis. Other etiologies were investigated, with electrophoresis and bone marrow aspiration, and subsequently excluded.

Although data on side effects of parenteral fosfomycin is scarce, 6 cases of leucopenia and 5 cases of neutropenia were reported, without details, in the literature [4, 6–8]. In the same way, the French summary of product characteristics mentions that cases of transient neutropenia and agranulocytosis have been described, without any further details on the reported cases [9]. The transient aspect needs to be further studied. An analysis of the FDA Adverse Event Reporting System Database reported a higher than expected frequency of agranulocytosis (n = 16). 25 cases of decreases of white blood cell count were described. This adverse effect has been reported to occur between 1 and 116 days (median: 11 days) after starting fosfomycin treatment. Other drugs that could induce bone marrow toxicities were associated to in all cases. No relation was found between the intensity of the cytopenia and the administered dose. Several cases happened after a single dose of the drug. Parenteral fosfomycin is often use in critically ill patients to treat multiresistant bacteria and this population usually receives many other medications (other antimicrobials, proton pump inhibitors…), so that fosfomycin is rarely the only suspected drug. The authors of the FDA analysis differentiated the profile of the reported adverse effects according to the route of administration, and neutropenia was one of the adverse effects associated with parenteral fosfomycin (from 15 non-comparative trials). DRESS syndrome was associated to the neutropenia in 6 cases, and isolated eosinophilia in 8 cases. The mechanisms underlying this toxicity remain unknown, and none has been suggested so far. In the French national pharmacovigilance database, 3 cases of neutropenia where fosfomycin is the only suspected drug are registered. Among these cases, the time to onset was 8, 20 and 28 days and the outcome was favorable after stopping the drug in 2 cases (time to regression not known; in the third case, the patient died but without any relation to the adverse effect).

## Conclusion

Although sparse data is reported, awareness should be raised about agranulocytosis induced by fosfomycin. The mechanisms of this hematological toxicity remain to be clarified.

### Electronic supplementary material

Below is the link to the electronic supplementary material.

Supplementary Material 1

## Data Availability

Raw data in the supplementary material.
